# Conserved Usage of Alternative 5′ Untranslated Exons of the *GATA4* Gene

**DOI:** 10.1371/journal.pone.0008454

**Published:** 2009-12-24

**Authors:** Séverine Mazaud Guittot, Marie France Bouchard, Jean-Philippe Robert-Grenon, Claude Robert, Cynthia G. Goodyer, David W. Silversides, Robert S. Viger

**Affiliations:** 1 Reproduction, Perinatal and Child Health, Centre de Recherche du Centre Hospitalier Universitaire de Québec, Quebec City, Canada; 2 Centre de Recherche en Biologie de la Reproduction (CRBR), Laval University, Quebec City, Canada; 3 Department of Animal Science, Laval University, Quebec City, Canada; 4 McGill University Health Centre-Montreal Children's Hospital Research Institute, Montreal, Canada; 5 Faculty of Veterinary Medicine, University of Montreal, St-Hyacinthe, Canada; 6 Department of Obstetrics and Gynecology, Laval University, Quebec City, Canada; University of Edinburgh, United Kingdom

## Abstract

**Background:**

GATA4 is an essential transcription factor required for the development and function of multiple organs. Despite this important role, our knowledge of how the *GATA4* gene is regulated remains limited. To better understand this regulation, we characterized the 5′ region of the mouse, rat, and human *GATA4* genes.

**Methodology/Principal Findings:**

Using 5′ RACE, we identified novel transcription start sites in all three species. *GATA4* is expressed as multiple transcripts with varying 5′ ends encoded by alternative untranslated first exons. Two of these non-coding first exons are conserved between species: exon 1a located 3.5 kb upstream of the GATA4 ATG site in exon 2, and a second first exon (exon 1b) located 28 kb further upstream. Expression of both mRNA variants was found in all GATA4-expressing organs but with a preference for the exon 1a–containing transcript. The exception was the testis where exon 1a– and 1b–containing transcripts were similarly expressed. In some tissues such as the intestine, alternative transcript expression appears to be regionally regulated. Polysome analysis suggests that both mRNA variants contribute to GATA4 protein synthesis.

**Conclusions/Significance:**

Taken together, our results indicate that the *GATA4* gene closely resembles the other GATA family members in terms of gene structure where alternative first exon usage appears to be an important mechanism for regulating its tissue- and cell-specific expression.

## Introduction

The GATA proteins are a small family of transcriptional regulators composed of 6 members that bind the common consensus sequence motif (A/T)GATA(A/G). GATA proteins share a highly homologous zinc finger DNA binding domain which is also highly conserved from invertebrates to mammals [1]. GATA family members display distinctive but overlapping expression patterns, both temporally and spatially. GATA1, 2, and 3 are primarily expressed in cells of the hematopoietic lineage (e.g., primitive erythroblasts, megakaryocytes, T cells, eosinophils), embryonic brain and liver, testis, and endothelial cells [2]–[4]. Conversely, GATA4, 5, and 6 are primarily detected in mesoderm- and endoderm-derived tissues such as the heart, lung, stomach, intestine, gonads, and blood vessels [3], [5], [6]. In agreement with these expression patterns, numerous GATA knockout models in mice as well as the *in vitro* identification of GATA target genes have revealed the crucial role of GATA factors for cellular differentiation during vertebrate organogenesis [2], [5], [6].

GATA4 is prominently expressed during embryogenesis in developing endoderm and mesoderm and their derivatives including the liver, intestine, stomach, pancreas, and gonads [7]–[14]. In the mouse, *Gata4* deficiency leads to early embryonic lethality due to deficiencies in the differentiation of the extraembryonic endoderm leading to misclosure of the ventral wall of the embryo and an inability to form a linear heart tube [15], [16]. Later studies, using tetraploid embryo complementation to rescue these lethal defects, revealed a definitive role for GATA4 in early cardiogenesis [17], and that GATA4, together with GATA6, is required for the onset of cardiac myocyte gene expression and differentiation [18]. The characterization of embryos generated from *Gata4* null ES cells by tetraploid complementation also revealed the essential role of GATA4 in early development of the liver and ventral pancreas [19]. In *Gata4* null embryos, the gut tube fails to close, again due to defects in visceral endoderm differentiation [15], [16]. Analysis of *Gata4* ^−/−^ chimeric mice showed that GATA4 is also required for proper differentiation of gastric epithelial cells [20]. Additional insights into the essential nature of GATA4 in stomach development has come from mice expressing a mutant form of GATA4 (*Gata* ^ki/ki^) that can no longer interact with its partner FOG2 [21]. The same mice were also used to demonstrate the role of GATA4 in early gonad development [22]–[24]. The ever-expanding list of GATA4 target genes have helped to define the pivotal role of this transcription factor in the molecular cascade of events in early organogenesis and proper organ function later in life [5], [6], [25]–[28].

Although many GATA4 target genes have been identified, and despite the importance of this factor in many developmental processes, our knowledge of how the *GATA4* gene is regulated remains surprisingly limited. Initial identification of *Gata4* transcripts was made by screening of cDNA libraries [7], [13]. The identified sequences included an untranslated first exon located 3.5 kb upstream of the ATG initiator codon in exon 2. We and others have begun to characterize the regulation of mouse *Gata4* transcription via regulatory sequences immediately upstream of this non-coding first exon [29], [30]. In both cardiac and gonadal cells, conserved GC boxes and an E-box element immediately upstream of its transcription initiation site appear to play an important role [29], [30].

Interestingly, the structural organization of the different vertebrate GATA genes shows several common features, including the use of multiple variably distant enhancers and the presence of alternative 5′ untranslated exons whose expression is controlled by distinct, and often tissue-specific, promoter units [31]–[37]. In the mouse, the *Gata4* gene is regulated by a least one distal enhancer required for early *Gata4* expression in lateral mesoderm and then eventually in the mesenchyme surrounding the fetal liver [38]. Since the expression of alternative transcripts differing in their 5′ ends is an important regulatory feature of many GATA genes [32], we hypothesized that this regulatory mechanism exists for the *GATA4* gene as well. Therefore, to gain further insights into the transcriptional regulation of the *GATA4* gene, we performed a detailed analysis of the 5′ ends of *GATA4* transcripts from different species generated by rapid amplification of cDNA ends-PCR (5′ RACE-PCR). We found that, indeed, the mouse, rat and human *GATA4* genes are expressed as multiple transcripts that differ in their first exon usage. Two of these exons, named E1a and E1b, are highly conserved between mammalian species and their differential usage likely contributes to the tissue- and/or cell-specific expression of the *GATA4* gene.

## Results

### The Mouse, Rat, and Human *GATA4* Genes Are Expressed as Multiple Transcripts with Alternative Untranslated First Exons

**Figure 1:**
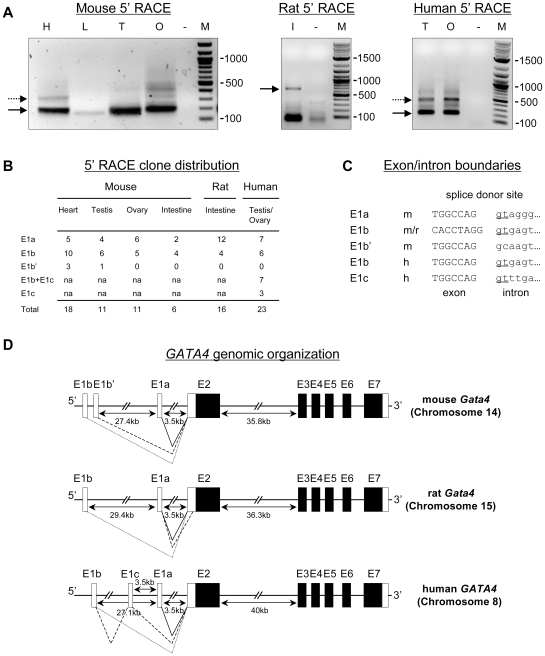
Identification of *GATA4* alternative transcripts. (A) Characterization of alternative 5′ *GATA4* mRNA ends by 5′ RACE of mouse, rat, and human transcripts obtained from heart (H), liver (L), testis (T), ovary (O), and intestine (I). Negative (water only) controls (−) and the base pair sizes of molecular weight markers (M) are also shown. Continuous arrows point out each major band while discontinuous arrows point out additional higher molecular weight bands. (B) Quantitative distribution of the 5′ RACE products subcloned in pCRII-TOPO cloning vector and subsequently sequenced; na: not applicable. (C) Sequences of the splice donor site of intron 1 of the mouse (m), rat (r) and human (h) *GATA4* genes. (D) Schematic representation of the genomic organization of previously known and newly identified untranslated first exons of mouse, rat and human *GATA4* loci. Continuous and dotted lines represent possible first exon associations. Intronic distances between 5′ untranslated first exons and between the first translated exons (E2) and E3 are indicated.

The initial characterization of *GATA4* transcripts showed that *GATA4* transcription begins with an untranslated first exon located approximately 3.5 kb upstream of its translation initiation site [7], [13]. However, an analysis of the genomic organization of the other mammalian GATA genes has revealed that they share several common features, including differential first exon usage driven by alternative upstream promoters [32]. Surprisingly, a similar property has not yet been described for the *GATA4* gene. A database search of *GATA4* nucleotide sequences, however, has revealed the presence of at least one EST from a human intestine cDNA library that shares partial homology to GATA4 but which differs from the classical sequence in its 5′ end (GenBank AK097060). This provides initial evidence that *GATA4* transcripts with variable 5′ termini likely exist. To verify that GATA4 is no exception to the 5′ untranslated exon rule, and to gain additional insights into the transcriptional mechanisms that regulate *GATA4* gene expression, we analyzed the 5′ ends of *GATA4* transcripts by 5′ RACE (Figure 1A and B). RACE reactions were carried out using RNA isolated from mouse, rat, and human tissue samples known to express GATA4 (Figure 1A). For the three species examined, the RACE reaction yielded a major band of similar size as visualized by electrophoresis in a standard agarose ethidium bromide-stained gel (Figure 1A, solid arrows). An additional higher molecular weight minor band was also evident for the mouse and human samples (Figure 1A, dashed arrows).

Surprisingly, sequence analysis of the products cloned from the major RACE band in mouse revealed that it contained not one but rather three distinct cDNAs that differed in their 5′ termini. We found clones containing the expected non-coding first exon of *Gata4* (renamed as E1a) located 3.5 kb upstream of E2 in all tissues examined (Figure 1B and D). *GATA4* E1a-containing transcripts were equally well represented in the rat and human samples (Figure 1B). We also identified *GATA4* transcripts containing two novel exons (named E1b and E1b′) approximately 30 kb distant from E2 (Figure 1D). The E1b′-containing transcript was the least abundant of the three RACE clones identified and was restricted to mouse heart and testis (Figure 1B). The heart also expressed a longer form of the E1b′ transcript (Figure 1A, dashed arrow in left panel). E1b-containing transcripts, however, were present in all tissues and all three species (Figure 1B).

RACE clones derived from human tissues samples revealed an additional first exon (named E1c) located midway between E1a and E1b (Figure 1D). In both human testis and ovary (Figure 1B), *GATA4* transcripts were identified that contained E1c alone (Figure 1A, solid arrow in right panel) or E1c joined to E1b (Figure 1A, dashed arrow). This differed from the mouse and rat where E1a, E1b, and E1b′ were used independently of one another.

With respect to sequence homology, E1a and E1b were less well conserved among species than were the other *GATA4* coding exons, especially E2 (Figure S1). Interestingly, E1b displayed a higher sequence conservation (94.4% between mouse and rat, 69% between mouse and human) than E1a (72.7% between mouse and rat, 59.8% between mouse and human). In agreement with the 5′ RACE clone distribution (Figure 1B), E1b′ and E1c were not conserved, and thus appear to be species-specific. Inspection of the exon/intron boundaries of the different first exons showed that the sequences at the splice donor sites conform to the AG-gt splice rule and represent either canonical (E1a, E1b, E1c) or non-canonical (E1b′) splice sites (Figure 1C). The large distance separating E1a and E1b (∼28 kb) was well conserved between species, as was the distance between E1 and E2 (∼3.5 kb) (Figures 1D and S1).

**Figure 2:**
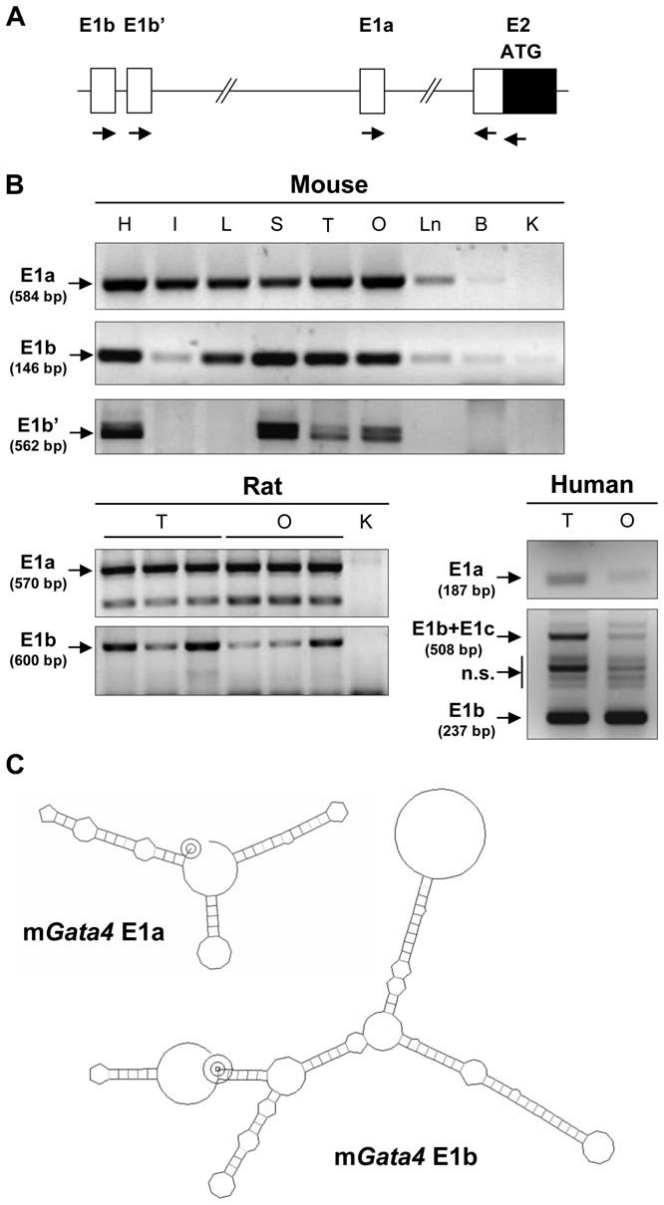
*GATA4* mRNA variant expression and secondary structure. (A) Location of primers used for PCR amplification of the *GATA4* mRNA variants; specific 5′ primers (indicated on the *GATA4* locus) were used in combination with a common 3′ primer. (B) RT-PCR amplification of *GATA4* E1a, E1b, and E1b′ mRNA variants in *GATA4*-expressing tissues (H, heart; I, intestine; L, liver; S, stomach; T, testis; O, ovary), and control tissues that weakly or do not express GATA4 (Ln, lung; B, brain; K, kidney). Tissues were obtained from adult mouse and rat and from fetal human samples. The identity of the *GATA4* mRNA variants amplified with the human samples was verified by cloning and sequencing the products. (C) Predicted secondary structures of the mouse *Gata4* E1a or E1b mRNA sequences. The structures were predicted using the RNA draw program [51]. Calculated free energy values for the structures were −33.59 kcal/mol for E1a and −58.59 kcal/mol for E1b. Start sites are indicated by concentric circles.

The identity and tissue distribution of the different *GATA4* transcripts were confirmed by conventional RT-PCR using exon 1-specific primers for each species as shown in Figure 2A. All PCR products were subcloned and sequenced to confirm the identity of the amplified transcripts. In mouse, *Gata4* transcripts containing E1a were present in all adult organs known to express GATA4 (Figure 2B). The same was true for E1b-containing transcripts with the exception of the intestine where PCR amplification was variable (i.e., some times strong, some times weak). *Gata4* transcripts containing E1b′ exhibited a more restricted pattern with expression detected as a doublet band (long and short forms) only in heart, stomach, and gonads (Figure 2B).

In the rat, *Gata4* transcripts containing either E1a or E1b were also amplified in GATA4-positive tissues such as the testis and ovary (Figure 2B). Interestingly, amplification with E1a-specific primers in rat tissues resulted in 2 bands. Sequencing showed that the higher molecular weight band was the expected transcript and the lower molecular weight band was an association between E1a and a shorter part of E2 (Figure 1D, dotted line).

In human tissues, we could amplify both *GATA4* E1a and E1b-containing transcripts (Figure 2B). E1b was amplified either alone or fused with a novel exon, E1c. The E1b-E1c amplified sequence matched one found in a human intestine EST database (GenBank AK097060), except that E1c in the EST sequence was connected to E3 and not E2 as in our clones. E1b-containing transcripts are likely to have a more complex secondary structure when compared to E1a transcripts as the calculated free energy value for the E1b sequence was almost twice as that of the E1a sequence (Figure 2C). Our conventional PCR studies therefore show that although *GATA4* E1b′ and E1c are species-specific, E1a and E1b are well conserved and are likely physiologically relevant to *GATA4* gene regulation in different tissues. In light of this, the remainder of the present study is focused on *Gata4* E1a and E1b transcripts in mouse tissues.

### *Gata4* Transcripts That Initiate with Either E1a or E1b Non-Coding Exons Are Widely and Coordinately Expressed in Fetal and Adult Mouse Tissues

**Figure 3:**
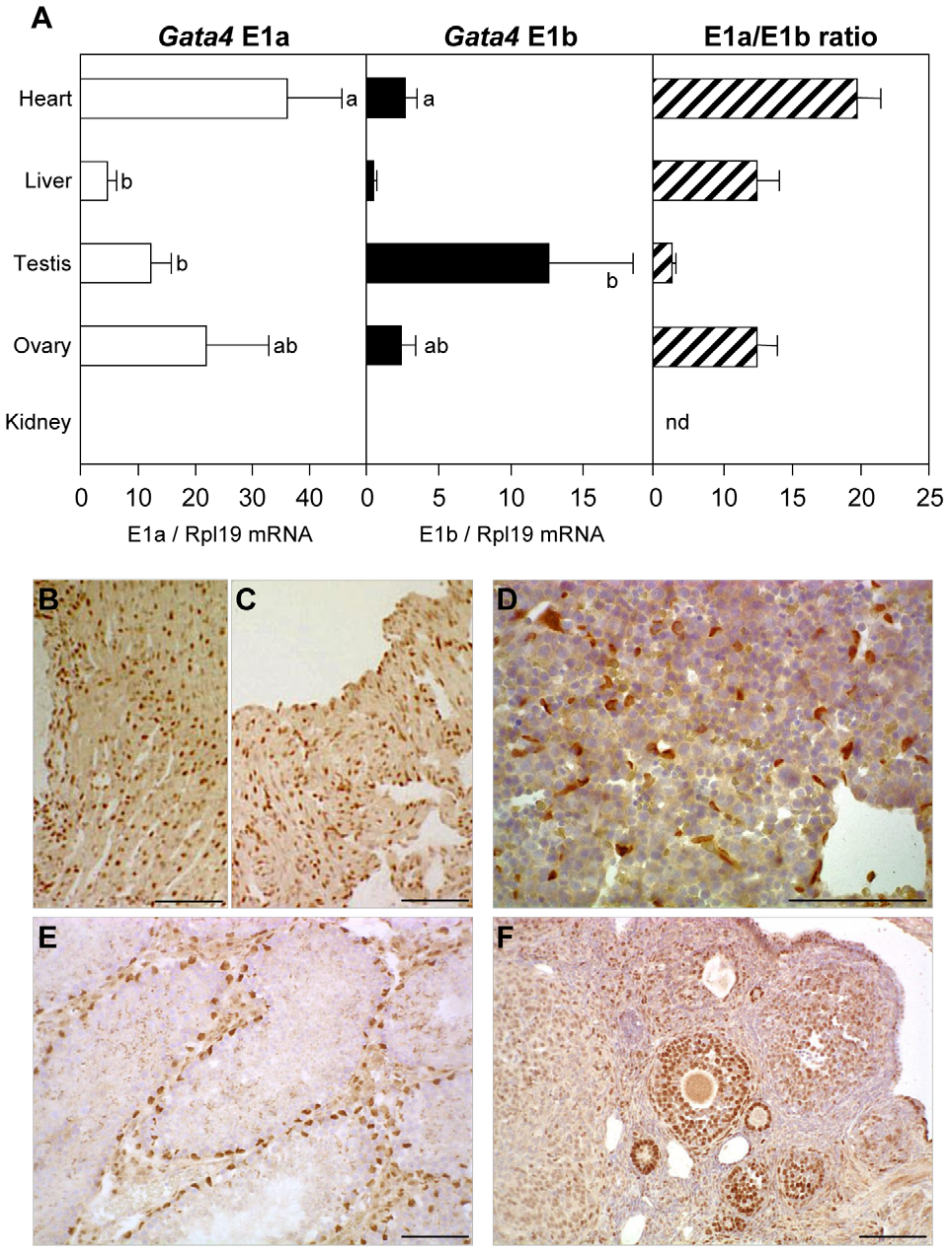
Quantitative expression of E1a- and E1b-containing *Gata4* transcripts in adult mouse organs. (A) Quantitative PCR (qPCR) was used to assess relative *Gata4* E1a and E1b mRNA levels in several organs known to robustly express GATA4; kidney was used as a negative control. Data are reported in arbitrary units as a ratio of the level of *Gata4* E1a or E1b mRNA variant to that of the *Rpl19* reference gene ±SEM. Groups with like letters are not significantly different. The E1a to E1b ratio (E1a level divided by E1b level) indicates the relative proportion of each mRNA in these organs and highlights the importance of the E1a transcript, except in the testis, where both the E1a and E1b *Gata4* mRNA variants are equally represented (ratio close to 1); nd, not defined. (B–F) Representative GATA4 immunohistochemistry in mouse adult heart atrium (B) and ventricle (C), liver (D), testis (E) and ovary (F) showing the relative proportion of *Gata4*-expressing cells in each organ. Scale bars: 100 µm.

In order to study the relative abundance of the different *Gata4* mRNA variants in adult mouse tissues, we performed qPCR on RNA derived from several *Gata4*-expressing organs (Figure 3). *Gata4* E1a mRNA levels were well correlated with the expected variation of *Gata4* expression in heart, liver and gonads (Figure 3A, left panel). Highest levels were found in heart and ovary, where the majority of the cells contain GATA4 protein (Figure 3B, C, and F). Lower levels of *Gata4* E1a transcripts were observed in liver and testis (Figure 3A, left panel), where only subpopulations of cells are GATA4-positive (Figure 3D and E). With the exception of the testis, *Gata4* E1b mRNA levels were about 10–20% of those observed for the E1a variant (Figure 3A, middle panel). A comparison of E1a vs. E1b transcript expression in the different tissues showed that the testis, with an E1a/E1b ratio of 1, robustly expresses the E1b mRNA variant (Figure 3A, right panel).

**Figure 4:**
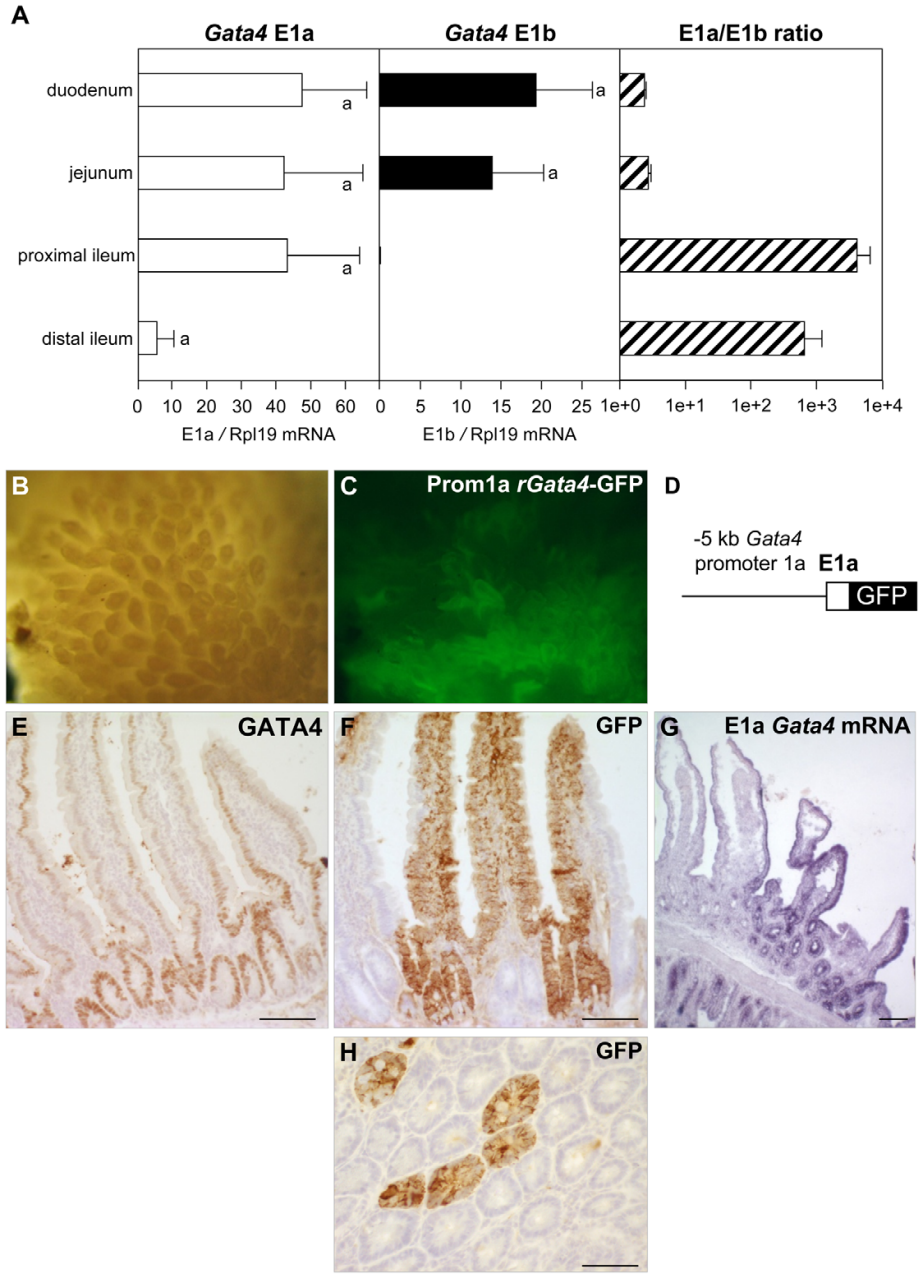
Quantitative expression of E1a- and E1b-containing *Gata4* transcripts in mouse intestine. (A) qPCR was used to assess relative *Gata4* E1a and E1b mRNA levels in successive antero-posterior intestine segments. Data are reported in arbitrary units as a ratio of the level of *Gata4* E1a or E1b mRNA variant to that of the *Rpl19* reference gene ±SEM. Groups with like letters are not significantly different. The E1a to E1b ratio (E1a level divided by E1b level) indicates the relative proportion of each mRNA in the different intestinal regions and reveals the increasing proportion of E1a *Gata4* mRNA compared to E1b *Gata4* mRNA going from the most anterior to the most posterior segments. (B–G) Heterogeneous expression of *Gata4* E1a transcripts in the intestine. Bright field (B) and fluorescent confocal view (C) of adult mouse intestine villosities expressing a GFP transgene under the control of 5 kb of proximal *Gata4* 1a promoter sequence (construct is shown in D). Immunohistochemistry for endogenous GATA4 protein (E). Detection of *Gata4* E1a transcripts by *in situ* hybridization (G) shows a patchy distribution pattern similar to the immunohistochemical detection of promoter 1a driven GFP expression viewed in either a longitudinal (F) or transverse section (H). Scale bars: 100 µm.

The intestine was not included in the initial qPCR analysis since this tissue exhibits a marked anterior-to-posterior decrease in *Gata4* expression [39], [40]. Expression of the *Gata4* E1a and E1b transcripts in this tissue was therefore studied as a function of intestinal segment (Figure 4A). *Gata4* E1a mRNA levels followed this classic anterior-to-posterior profile (Figure 4A, left panel). *Gata4* E1b mRNA levels had a similar profile but with a sharp decrease between the jejunum and ileum (Figure 4A, middle panel). The E1a/E1b ratio showed that the transcripts were expressed at a similar level in the anterior part of the tissue (Figure 4A, right panel). In the more distal segments, this E1a/E1b ratio was not informative due to the absence of E1b transcripts. This is likely the reason for the variable detection of the E1b transcript in our conventional PCR amplifications (Figure 2B). To better understand *Gata4* mRNA variant expression in the intestine, we made use of our previously reported transgenic mouse line [29], that expresses a GFP fluorescent reporter under the control of *Gata4* regulatory sequences immediately upstream of E1a (the −5 kb *Gata4* E1a promoter-GFP construct is shown in Figure 4D). Macroscopic views of adult intestine revealed a patchy pattern of expression of the GFP transgene (Figure 4B and C). This was similar to what we observed for *Gata4* E1a mRNA detected by *in situ* hybridization using an E1a-specific probe (Figure 4G). Immunohistochemistry analysis confirmed that whereas GATA4 protein was present in all enterocytes of all villosities, with a crypt to apex gradient (Figure 4E), the GFP transgene (Figure 4F and H), much like the *Gata4* E1a mRNA (Figure 4G), was expressed in enterocytes of only a subset of villosities. Our data therefore show that both first exons are expressed in a coordinate fashion in *Gata4*-expressing adult organs, with the exception of the lower intestinal tract.

**Figure 5:**
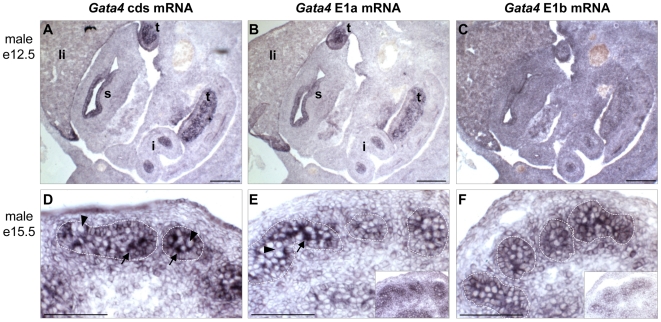
*In vivo* detection of *Gata4* E1a and E1b mRNA transcripts. *In situ* hybridization of *Gata4* mRNA with a riboprobe corresponding to the whole *Gata4* coding sequence (cds) (A, D), *Gata4* E1a (B, E), and *Gata4* E1b (C,F) transcripts in mouse male e12.5 embryos (A–C), and male e15.5 testes (D–F). Control E1a and E1b sense probes in male e15.5 testes are shown in the insets to (E) and (F), respectively. An obvious *Gata4* signal is present in testes (t), stomach epithelium (s), intestine epithelium (i), and in liver (li) of e12.5 male embryos with the *Gata4* cds and *Gata4* E1a probes. An obvious *Gata4* signal (arrows) is also present in Sertoli cells of testicular cords (areas enclosed by dotted lines) of e15.5 male testes with the *Gata4* cds and *Gata4* E1a probes. Unreactive germ cells are indicated by arrowheads. Scale bars: 100 µm.

We further investigated the pattern of *Gata4* E1a and E1b transcript expression by *in situ* hybridization in mouse fetal organs (Figure 5). We compared the pattern of expression of all *Gata4* transcripts using a coding sequence (cds) riboprobe to exon 1-specific *Gata4* transcripts using E1a and E1b-specific riboprobes. As expected, using a probe encompassing the whole coding sequence from E2 to E7, which is a sequence shared by all *Gata4* transcripts, we found strong expression in testis cords, hindgut and intestinal epithelia, differentiating pancreas and hepatic mesenchyme at e12.5 (Figure 5A). In the e15.5 testis, global *Gata4* transcripts were found in Sertoli cells inside cords, and in scattered cells in the mesenchyme that corresponded to fetal Leydig cells, and also in the thin epithelium covering the tunica albuginea, reminiscent of the coelomic epithelium (Figure 5D). Using a mouse E1a-specific riboprobe, we observed an expression profile identical to, albeit at a lower level, that observed using the *Gata4* cds riboprobe (Figure 5B and E). The use of E1b-specific probes was unsuccessful in *in situ* analysis both in fetal (Figure 5C and F) and adult organs (data not shown). The low signal for the E1b riboprobe was difficult to distinguish from background and the non-specific signal caused by the sense probes (insets to Figure 5E and F). Our inability to obtain a E1b-specific signal might be explained by the more complex secondary structure of the E1b mRNA sequence (Figure 2C), that could have impeded the hybridization reaction. It might also be due to the lower level of expression of the E1b transcript as we observed in our qPCR analysis of adult organs (Figures 3A and 4A). The *Gata4* E1b transcript might also be absent in the fetal testis despite its strong expression in the adult testis (Figure 3A). Although the E1b *in situ* experiments were inconclusive, our E1a *in situ* data demonstrate that *Gata4* E1a transcripts are at least expressed in a similar pattern to that of the *Gata4* gene.

### *Gata4* E1a and E1b Transcripts Are Actively Translated

**Figure 6:**
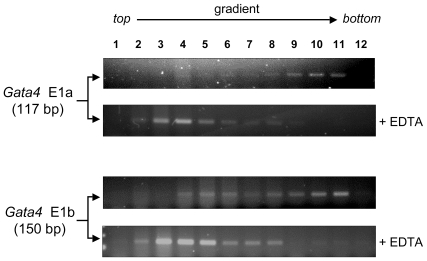
Polysome analysis of *Gata4* E1a and E1b transcripts in the testis. RNA from adult mouse testis was centrifuged through a linear sucrose gradient to separate transcripts associated with monoribosomes (not actively translated) and polyribosomes (actively translated). Monoribosomal and polyribosomal fractions are found at the top and towards the bottom of the gradient, respectively. Twelve fractions were collected from the gradient, the RNA was extracted from each fraction, reverse-transcribed into cDNA and subjected to PCR with primers specific for *Gata4* E1a and E1b. Both transcripts are associated with the polyribosomal fractions indicating that they are being actively translated into protein. EDTA, which sequesters magnesium ions required for ribosome stability, was used as a control. In the presence of EDTA, the transcript profiles show a characteristic shift to the top of the gradient indicating a successful dissociation of the polyribosomes.

Since the *Gata4* alternative first exons are non-coding, an immunohistochemical approach using isoform-specific antibodies could not be used to assess the biological relevance of the E1a and E1b mRNA variants. To demonstrate that the two *Gata4* mRNA variants are both likely translated into protein, we performed a polysome analysis of the E1a and E1b transcripts in the adult mouse testis (Figure 6). In this assay, a sucrose gradient is used to separate mRNA species associated with monoribosomes located at the top of the gradient (mRNAs not actively translated) vs. those associated with polyribosomes at the bottom of the gradient (mRNAs actively translated). As shown in Figure 6, the *Gata4* E1a and E1b transcripts (detected by conventional PCR) were associated with the heaviest (polyribosomal) fractions located at the bottom of the gradient indicating that both transcripts are actively translated into protein. A control experiment, where EDTA was added to the testis samples and the sucrose gradient to dissociate the polyribosomes, was included to demonstrate that the *Gata4* E1a and E1b mRNAs were indeed associated with the polyribosomal fractions.

## Discussion

GATA4 is a member of the GATA family of transcriptional regulators that is required for the development and differentiation of numerous organs in the early embryo. This pivotal developmental role emphasizes the importance of understanding the molecular mechanisms that regulate expression of the *GATA4* gene. Despite its critical role in organogenesis, our knowledge of *GATA4* gene transcription remains surprisingly limited. Here, we report the identification of novel *GATA4* transcripts that differ solely in their non-coding first exons. These alternative first exons are located several kilobases upstream of the classic *GATA4* transcription initiation site suggesting that their expression is being driven from novel upstream promoters. The fact that some of these first exons are species-conserved also indicates that alternative promoter usage is likely an important regulatory mechanism for controlling the tissue- and cell-specific expression of the *GATA4* gene in humans and other mammalian species.

As for the other GATA genes, the GATA4 ATG initiator codon and the start of its open reading frame is located in E2. Its originally described first exon, which is non-coding, is located 3.5 kb upstream of E2. Other GATA gene family members, however, are known to express multiple transcripts with different untranslated first exons (reviewed in [32]). Moreover, many of these alternative GATA transcripts are expressed in a tissue-specific manner. To determine whether GATA4 is or is not an exception to alternative first exon usage, we used 5′ RACE to screen GATA4 transcripts in mouse, rat, and human tissues. Our results confirmed the existence of additional first exons for the *GATA4* gene, located far upstream of E2 (summarized in Figure 1D). Conventional RT-PCR amplifications followed by sequencing of the PCR products showed that the novel GATA4 first exons are used alternatively and rarely in association with one another, the one exception being in the human (Figure 2B). All of the first exons are devoid of a translational start point and, thus, will not affect, the open reading frame coding for the GATA4 protein. Therefore our data show that the *GATA4* gene also exhibits prominent alternative first exon usage, a feature common to the other GATA genes [32], as well as many other genes that display complex temporal and/or spatial expression patterns [41].

Amongst the newly identified first exons, although E1b was well conserved between rodents and human, E1b′ and E1c were not. E1b′-containing transcripts were also expressed at very low levels as determined by qPCR (data not shown). In human tissues, we amplified transcripts containing E1c intercalated between E1b and E2. This is similar to a published EST (AK097060) where E1c is present but connected to E3. For these specific *GATA4* transcripts, the combination of poor conservation and low level of expression render the biological significance of these transcripts unlikely, as suggested for the *Nr5a1/Sf1* gene [42]. In contrast, E1b was not only well conserved between rodents and human from a sequence standpoint, but was also expressed at significant levels in most, if not all, GATA4-expressing tissues examined (Figures 3A and 4A). This was particularly true for the testis where E1a and E1b mRNA levels were comparable. Surprisingly, a previous study by Ohara *et al* [30] did not identify the E1b transcript in their 5′ RACE screen of mouse heart and testis. This might simply be due to differences in experimental procedures as the energy require to denature the secondary structure of E1b is predicted to be about twice as high as that needed for E1a (Figure 2C). At least in the heart, it might also be a reflection of the lower abundance of the E1b transcript. Although the E1b sequence appears to be conserved for mammalian *GATA4* genes, this may not extend to all vertebrate species. For example, an analysis of transcription initiation sites in the zebrafish *gata4* gene, has revealed that its ATG initiator codon is located in its first exon [43], thus ruling out the possibility of similar mammalian-like alternative 5′ untranslated exons. The absence of alternative first exon usage has also been reported for the chick *Gata6* gene and the zebrafish and Xenopus *Gata2* genes when compared to their orthologous mouse loci [32]. Consequently, the decrease in conservation of alternative 5′ leader exons with species divergence is not unique to GATA4.

While differential first exon usage is common to the mammalian GATA genes, their functional significance is less clear. For the hematopoietic GATA1/2/3 subfamily, promoter sequences upstream of different non-coding first exons have been shown to contribute to the tissue-specific expression of the factors. For example, regulatory sequences upstream of exon 1T direct *Gata1* expression specifically in the testis whereas sequences upstream of exon 1E direct *Gata1* expression in blood cells [33]. For the *GATA4* gene, however, the use of alternative first exons appears to be less well correlated with tissue specificity. Indeed, we detected *Gata4* E1a- and E1b-containing transcripts in several *GATA4*-expressing organs both in rodents and humans (Figure 2B). This suggests that both transcript variants are expressed in all GATA4-positive cell types or that they are specific for a cell type in a given organ. In the adult mouse intestine, where E1a is predominantly expressed, the pattern of GFP transgene expression directed by promoter 1a sequences correlated well with the *in situ* hybridization signal specific for this exon, but did not exactly match with the endogenous protein localization (Figure 4A–H). Indeed, GFP staining, as well as E1a mRNA, were present in a majority of epithelial cells but only in a subset of villosities whereas GATA4 protein could be detected in all villosities. Thus in the intestine where the epithelium is composed predominantly of enterocytes, we can speculate that E1a and E1b transcripts complement one another at a local level. Our demonstration that the *Gata4* E1a and E1b variants are translationally active (Figure 6) supports this hypothesis. However, in more complex organs such as the gonads which are composed of different cell types that express GATA4 [6], this picture is less clear due to our inability to obtain a reliable *in situ* hybridization signal for the E1b-specific transcript. As a result, other approaches such as generation of transgenic mice expressing a reporter gene driven by E1b promoter sequences will undoubtedly have to be used to answer this question.

It is interesting to note that GATA4 more closely resembles GATA6 than the hematopoietic GATA factors not only at the level of coding sequence but also in terms of its transcriptional regulation. Thus while differential exon 1 usage clearly contributes to tissue specificity in the case of the hematopoietic GATA1/2/3 subfamily [32], the mostly coordinated exon 1 usage for both GATA4 (this study) and GATA6 [31] suggests other roles. For GATA4, redundant transcripts could provide a backup mechanism to guarantee the availability of GATA4 protein in cell types where its presence is crucial for cell function, such as differentiation of Sertoli cells in the fetal testis [23], [24]. The promoter regulatory sequences driving expression of the different *GATA4* transcripts could be under the influence of distinct trans-regulatory factors and this may account for basal and/or induced *GATA4* expression in specific cell types. Alternatively, redundant transcripts might be required to maintain a high level of protein translation. Finally, the presence of different first exons may play regulatory roles themselves on either RNA stability, protein translation efficiency, or both, as it has recently been described for the *Cyp19* gene [44]. Because GATA4 function is paramount for vertebrate organogenesis, and because its dysregulation is increasingly being linked to human disease [45], further studies on the intrinsic properties of each first exon as well as the putative promoter sequences upstream of E1b will be of great interest to better understand the complex tissue specification of the *GATA4* gene.

## Materials and Methods

### Ethics Statement

Experiments involving mice or rats were performed following Canadian Council of Animal Care (CCAC) guidelines for the care and manipulation of animals used in medical research. Protocols involving the manipulation of animals were approved by the institutional ethics committees of the University of Montreal (comité d'éthique de l'utilisation des animaux, CÉUA) and Laval University (comité de protection des animaux, CPAUL). Animals were maintained in ventilated cages under standard conditions of 12 hours of light, 12 hours of dark, with water and commercial rodent chow provided *ad lib*.

For experiments involving human tissue samples, McGill University Health Centre Research ethics boards approved the studies and informed written consent was obtained in each case.

### Collection and Processing of Animal Tissues

Tissues from adult CD-1 mice and Sprague-Dawley rats were obtained from animals available on site that were euthanized by CO_2_ inhalation. Generation of the 5 kb rat *Gata4* promoter 1a-GFP transgenic mouse line has been described previously [29]. For mouse embryo studies, noon on the day that a vaginal plug was observed was designated as e0.5, and for postnatal studies, the day of birth was designated as P0. Dissected adult organs or embryos were immediately observed under a standard fluorescence stereomicroscope for GFP expression in transgenic animals and/or processed for further morphological analysis or placed in RNAlater solution (Ambion) and stored at −80°C for subsequent RNA extraction. For immunohistochemical analyses and *in situ* hybridizations, embryos or organs were fixed in 4% paraformaldehyde-PBS (pH 7.2) for 1 h to overnight at 4°C. Fixed tissues were then embedded in paraffin, cut into 7 µm-thick sections, and mounted on superfrost slides, or cryoprotected in 18% sucrose in PBS, embedded in Tissue-Tek OCT compound (Miles), cut into 8 µm-thick sections and mounted onto 3-aminopropyltriethoxysilane (Sigma)-treated glass slides and stored at −20°C.

### Human Tissues

Testicular and ovarian fetal tissue samples (16 and 19 weeks fetal age) were obtained after therapeutic abortion [46]. McGill University Health Centre Research ethics boards approved the studies and informed written consent was obtained in each case. Tissues were flash frozen and stored at −80°C until analysis.

### 5′ RACE

Transcriptional start sites of the *GATA4* gene were mapped by rapid amplification of cDNA ends (5′ RACE) using the GeneRacer kit according to instructions supplied by the manufacturer (Invitrogen). Primers used for the 5′ RACE were generated against exon 2 (E2): one just downstream of the translation initiation start site and the other, a nested primer located at the beginning of E2 (Table 1). Total cellular RNA used in the 5′ RACE experiments was isolated from different adult mouse and rat tissues using Trizol reagent according to the manufacturer's recommendations (Invitrogen). RNA from human testis and ovary samples was isolated using an RNeasy Plus extraction kit (Qiagen). Complementary DNA (cDNA) was obtained from 5 µg total RNA using random hexamers and Superscript III reverse transcriptase provided with the GeneRacer kit. 5′ RACE conditions were as follows: 2 min at 95°C, 5 cycles (95°C for 30 sec, 72°C for 45 sec), 25 cycles (95°C for 30 sec, 65°C for 30 sec, 72°C for 45 sec), and a final extension of 10 min at 72°C. The reaction product was then used in a second round of PCR using the same conditions. Amplified products from the two rounds of nested PCR using the GATA4-specific primers (Table 1) were subcloned into pCRII-TOPO cloning vector (Invitrogen) and 46, 16, and 23 individual clones were sequenced from mouse, rat and human samples, respectively. All novel GATA4 sequences were deposited in the GenBank database — mouse: DQ436912, DQ436913, DQ436914, DQ436915; rat: DQ436916, and human: FJ169610. Sequence homologies between the different untranslated exons were analyzed using the ISHAN sequence homology analysis package [47].

**Table 1:** Oligonucleotide primers used in this study.

Primer use	Species	Sequence
5′ RACE (start of exon 2)	m/r	R: 5′-ACCAGAGCGGCTCCAGCGAA-3′
5′ RACE (start of exon 2)	h	R: 5′-CACAGGCGCAGAAGCTGCTA-3′
5′ RACE/PCR/qPCR/ISH *Gata4* reverse primer (exon 2 after ATG)	m/r/h	R: 5′-CGTGGTTGGCGGCCATGGCCAGGCT-3′
PCR *Gata4* reverse primer (exon 2)	h	R: 5′-GCGCACCTATTGGGGGCAGAAGAC-3′
PCR/qPCR/ISH *Gata4* (exon 1a)	m/r	F: 5′-TCCGCGGACTCACGGAGATC-3′
PCR *Gata4* (exon 1a)	h	F: 5′- GCGGAACCTCCAGGCCCAGCAGG-3′
PCR/qPCR/ISH *Gata4* (exon 1b)	m/r	F: 5′-ACAGGCTGGAATCTCTGGGCCT-3′
PCR *Gata4* (exon 1b)	h	F: 5′-CTGGAATCCCTGTGCAGAGTTTGCC-3′
PCR *Gata4* (exon 1b′)	m	F: 5′-GCTCCCTCCAGCAAACCCAGAT-3′
PCR/qPCR *Rpl19*	m	F: 5′-CTGAAGGTCAAAGGGAATGTG-3′
		R: 5′-GGACAGAGTCTTGATGATCTC-3′

m, mouse; r, rat; h, human; F, forward primer; R, reverse primer; ISH, *in situ* hybridization.

### Conventional and Quantitative PCR

Conventional PCR was done using Vent polymerase (New England Biolabs) and a Mastercycler gradient thermocycler (Eppendorf) under the following conditions—3 min at 95°C followed by 30 cycles of: denaturation (30 sec at 95°C), annealing (30 sec at 60°C), extension (30 sec at 72°C), with a final extension of 5 min at 72°C. PCR products were then analyzed by agarose gel electrophoresis and ethidium bromide staining. Primers used for amplification of each alternative first exon are shown in Table 1. For mouse and rat tissues, each amplification was performed three times using three different preparations of first strand cDNAs resulting from three different RNA extractions. Due to the limited quantity and availability of the human material, amplifications were done on one sample but from two different tissues (testis and ovary). In both cases, the amplified products were found to be identical as verified by sequencing.

For quantitative PCR (qPCR), total RNA was isolated from mouse tissues as described above. First strand cDNAs were synthesized from a 2-µg aliquot of the various RNAs using the Superscript II Reverse Transcriptase System (Invitrogen). Real time PCR was performed using a LightCycler 1.5 instrument and the LightCycler FastStart DNA Master SYBR Green I kit (Roche Diagnostics) according to the manufacturer's protocol. Primers used for qPCR are shown in Table 1. All qPCR runs were done using the following conditions: 10 min at 95°C followed by 35 cycles of denaturation (5 sec at 95°C), annealing (5 sec at 62°C), and extension (20 sec at 72°C) with a single acquisition of fluorescence levels at the end of each extension step. Each amplification was performed in duplicate using at least three different preparations of first-strand cDNAs prepared from each organ (n≥3). The specificity of the amplified PCR products was confirmed by analysis of the melting curve and agarose gel electrophoresis. Differences in mRNA levels between samples were quantitated using the standard curve method. DNA fragments containing E1a and E1b of the mouse *Gata4* gene were amplified by PCR and cloned into pGEM-T easy vector (Promega) to generate the E1a and E1b standards for preparing the dilution curves. A cloned fragment of the ribosomal gene *Rpl19* served as reference gene [48]. Serial dilutions of the target and reference plasmids ranging from 10^−4^ (0.1 ng/ml) to 10^−7^ (0.1 pg/ml) were prepared to generate the standard curves. The amount of DNA for the target (E1a, E1b) and reference (*Rpl19*) in the unknown samples was calculated by the LightCycler software 3.5 (Roche Diagnostics) using the respective dilution curves. Data are reported in arbitrary units as a ratio of the level of *Gata4* E1a or E1b mRNA variant in each sample to that of the *Rpl19* reference gene.

### *In situ* Hybridization

Specific mouse *Gata4* E1a and E1b cDNAs were generated by PCR and cloned into pGEM-T easy vector; the corresponding primers are provided in Table 1. A pcDNA3-GATA4 plasmid encoding the entire GATA4 coding sequence was used as a control probe [49]. Digoxigenin-labeled riboprobes were generated by *in vitro* transcription using a DIG RNA labeling kit (Roche Diagnostics). *In situ* hybridization on frozen tissue sections was carried out as previously described [50].

### Immunohistochemistry

Following paraffin removal and rehydration, endogenous peroxidase activity was blocked with 0.3% hydrogen peroxide in methanol for 30 min. Frozen tissue sections were thawed, delipidized in chloroform, rehydrated in PBS, and endogenous peroxidase activity was blocked with 3% hydrogen peroxide for 10 min. For GFP and GATA4 immunodetection, sections were boiled for 5 min in 0.1M citrate buffer (pH 6.0) for antigen retrieval, blocked with 10% horse serum (in PBS with 8% BSA) for at least 20 min, and finally incubated overnight at 4°C with primary antibody diluted in blocking solution (PBS containing 0.1% BSA). Primary antibodies were directed against GATA4 (diluted 1/1000, cat. # sc-1237X, Santa Cruz Biotechnology) and GFP (diluted 1/2000, cat. # RDI-GRNFP4abr, RDI division of Fitzgerald Industries Intl.). After washing in PBS, sections were incubated for 45 min with either horseradish peroxidase-conjugated anti-rabbit antibody (Dako) or biotinylated anti-goat antibody (1/500 dilution; Vector Laboratories) and for 30 min with a peroxidase-conjugated streptavidin-horseradish complex (LSAB+ Kit; Dako) for GATA4. The reaction product was developed using 3,3′-diaminobenzidine tetrahydrochloride (Dako). Sections were counterstained with hematoxylin and mounted with glycerol-gelatin (Sigma). For negative controls, primary antibody was omitted and this generated no visible reaction. Slides were analyzed with a Zeiss Akioskop II epifluorescence microscope (Carl Zeiss) connected to a digital camera (Spot RT Slider, Diagnostic Instruments).

### Polysome Analysis

Testes were collected from SV129 adult male mice immediately after euthanasia and individually pulverized in liquid nitrogen using a mortar. For each sample, the powder was lysed in 200 µl of polysome lysis buffer (PLB) containing 50 mM Tris pH 8.7, 150 mM KCl, 1.25 mM MgCl_2_, 1% IGEPAL, 0.5% deoxycholate, 10 mM DTT, 100 µg/ml cycloheximide, 1000 U/ml Protector RNase inhibitor (Roche Diagnostics) and supplemented with EDTA-free Mini Complete protease inhibitor cocktail (Roche Diagnostics). A control sample was made by adding EDTA (100 mM) to the PLB as well as to the sucrose gradient solution. The presence of EDTA sequesters the magnesium ions required for ribosome stability. Samples were disrupted by vortexing twice for 10 sec, intermitted by a 30 sec pause and clarified by a 20 min centrifugation at 12,000 g. Supernatants were assayed for absorbance at 260 nm using a NanoDrop ND-1000 spectrophotometer (Thermo Scientific). Samples (35 OD units) were loaded on a 4 ml 15-45% linear sucrose gradient and centrifuged for 3 h at 34,000 rpm in a SW 60 Ti rotor (Beckman Coulter). Fractionation was carried out by continuous absorbance monitoring at 254 nm using the BR-188 Density Gradient Fraction System (Brandel). Twelve fractions of approximately 350 µl were collected from the gradient using a Foxy 200 automated fraction collector (Teledyne Isco) and directly added to 428 µl of 5.25 M guanidinium thiocyanate. Fractions were precipitated overnight at −20°C by adding 2 µl of linear acrylamide (Ambion) used as carrier and 778 µl of 100% ethanol. Fractions were spun at 10,000 g for 20 min at 4°C, washed with 85% ethanol, and spun again at 10,000 g for 20 min. Once the supernatant was discarded, the RNA from each pellet was further purified using the Picopure RNA isolation kit (Molecular Devices). An aliquot of each fraction was used to quantify and assess the integrity of RNA using the 2100 Bioanalyser RNA 6000 Nano Chip kit (Agilent Technologies). The remaining total RNA content of each faction was reversed transcribed using SuperScript II (Invitrogen) following the manufacturer's protocol. The reaction was primed using 50 µM of oligo-dT(18). Fractions were then assayed for the presence of *Gata4* E1a or E1b by conventional PCR as described above.

### Statistical Analysis

Statistical analyses were done using the nonparametric Kruskal-Wallis one-way analysis of variance followed by Mann-Whitney U-tests to identify significant differences between groups; a P value less than 0.05 was considered significant. All statistical analyses were done using the SigmaStat software package (Systat Software).

## Supporting Information

Figure S1 MultiPipMaker [52] sequence comparison of GATA4 rat (used as reference sequence), mouse, human and dog loci. The percent sequence identity per 100 consecutive bp is indicated on the right. Exons are boxed in blue; highly conserved non-coding regions are highlighted by a green oval.
(1.63 MB TIF)

## References

[1] Lowry JA, Atchley WR (2000). Molecular evolution of the GATA family of transcription factors: conservation within the DNA-binding domain.. J Mol Evol.

[2] Weiss MJ, Orkin SH (1995). GATA transcription factors: key regulators of hematopoiesis.. Exp Hematol.

[3] Patient RK, McGhee JD (2002). The GATA family (vertebrates and invertebrates).. Curr Opin Genet Dev.

[4] Harigae H (2006). GATA transcription factors and hematological diseases.. Tohoku J Exp Med.

[5] Molkentin JD (2000). The zinc finger-containing transcription factors GATA-4,-5, and -6. Ubiquitously expressed regulators of tissue-specific gene expression.. J Biol Chem.

[6] Viger RS, Mazaud Guittot S, Anttonen M, Wilson DB, Heikinheimo M (2008). Role of the GATA family of transcription factors in endocrine development, function, and disease.. Mol Endocrinol.

[7] Arceci RJ, King AA, Simon MC, Orkin SH, Wilson DB (1993). Mouse GATA-4: a retinoic acid-inducible GATA-binding transcription factor expressed in endodermally derived tissues and heart.. Mol Cell Biol.

[8] Heikinheimo M, Scandrett JM, Wilson DB (1994). Localization of transcription factor GATA-4 to regions of the mouse embryo involved in cardiac development.. Dev Biol.

[9] Ketola I, Rahman N, Toppari J, Bielinska M, Porter-Tinge SB (1999). Expression and regulation of transcription factors GATA-4 and GATA-6 in developing mouse testis.. Endocrinology.

[10] Ketola I, Otonkoski T, Pulkkinen MA, Niemi H, Palgi J (2004). Transcription factor GATA-6 is expressed in the endocrine and GATA-4 in the exocrine pancreas.. Mol Cell Endocrinol.

[11] Kiiveri S, Liu J, Westerholm-Ormio M, Narita N, Wilson DB (2002). Differential Expression of GATA-4 and GATA-6 in Fetal and Adult Mouse and Human Adrenal Tissue.. Endocrinology.

[12] Nemer G, Nemer M (2003). Transcriptional activation of BMP-4 and regulation of mammalian organogenesis by GATA-4 and -6.. Dev Biol.

[13] Tamura S, Wang XH, Maeda M, Futai M (1993). Gastric DNA-binding proteins recognize upstream sequence motifs of parietal cell-specific genes.. Proc Natl Acad Sci U S A.

[14] Viger RS, Mertineit C, Trasler JM, Nemer M (1998). Transcription factor GATA-4 is expressed in a sexually dimorphic pattern during mouse gonadal development and is a potent activator of the Müllerian inhibiting substance promoter.. Development.

[15] Kuo CT, Morrisey EE, Anandappa R, Sigrist K, Lu MM (1997). GATA-4 transcription factor is required for ventral morphogenesis and heart tube formation.. Genes Dev.

[16] Molkentin JD, Lin Q, Duncan SA, Olson EN (1997). Requirement of the transcription factor GATA-4 for heart tube formation and ventral morphogenesis.. Genes Dev.

[17] Watt AJ, Battle MA, Li J, Duncan SA (2004). GATA4 is essential for formation of the proepicardium and regulates cardiogenesis.. Proc Natl Acad Sci U S A.

[18] Zhao R, Watt AJ, Battle MA, Li J, Bondow BJ (2008). Loss of both GATA4 and GATA6 blocks cardiac myocyte differentiation and results in acardia in mice.. Dev Biol.

[19] Watt AJ, Zhao R, Li J, Duncan SA (2007). Development of the mammalian liver and ventral pancreas is dependent on GATA4.. BMC Dev Biol.

[20] Jacobsen CM, Narita N, Bielinska M, Syder AJ, Gordon JI (2002). Genetic mosaic analysis reveals that GATA-4 is required for proper differentiation of mouse gastric epithelium.. Dev Biol.

[21] Jacobsen CM, Mannisto S, Porter-Tinge S, Genova E, Parviainen H (2005). GATA-4:FOG interactions regulate gastric epithelial development in the mouse.. Dev Dyn.

[22] Bouma GJ, Washburn LL, Albrecht KH, Eicher EM (2007). Correct dosage of Fog2 and Gata4 transcription factors is critical for fetal testis development in mice.. Proc Natl Acad Sci U S A.

[23] Manuylov NL, Fujiwara Y, Adameyko II, Poulat F, Tevosian SG (2007). The regulation of Sox9 gene expression by the GATA4/FOG2 transcriptional complex in dominant XX sex reversal mouse models.. Dev Biol.

[24] Tevosian SG, Albrecht KH, Crispino JD, Fujiwara Y, Eicher EM (2002). Gonadal differentiation, sex determination and normal Sry expression in mice require direct interaction between transcription partners GATA4 and FOG2.. Development.

[25] LaVoie HA (2003). The role of GATA in mammalian reproduction.. Exp Biol Med.

[26] Peterkin T, Gibson A, Loose M, Patient R (2005). The roles of GATA-4, -5 and-6 in vertebrate heart development.. Semin Cell Dev Biol.

[27] Tremblay JJ, Viger RS (2003). Novel roles for GATA transcription factors in the regulation of steroidogenesis.. J Steroid Biochem Mol Biol.

[28] Viger RS, Taniguchi H, Robert NM, Tremblay JJ (2004). Role of the GATA family of transcription factors in andrology.. J Androl.

[29] Mazaud Guittot S, Tetu A, Legault E, Pilon N, Silversides DW (2007). The proximal Gata4 promoter directs reporter gene expression to sertoli cells during mouse gonadal development.. Biol Reprod.

[30] Ohara Y, Atarashi T, Ishibashi T, Ohashi-Kobayashi A, Maeda M (2006). GATA-4 gene organization and analysis of its promoter.. Biol Pharm Bull.

[31] Brewer A, Gove C, Davies A, McNulty C, Barrow D (1999). The human and mouse GATA-6 genes utilize two promoters and two initiation codons.. J Biol Chem.

[32] Burch JB (2005). Regulation of GATA gene expression during vertebrate development.. Semin Cell Dev Biol.

[33] Onodera K, Yomogida K, Suwabe N, Takahashi S, Muraosa Y (1997). Conserved structure, regulatory elements, and transcriptional regulation from the GATA-1 gene testis promoter.. J Biochem.

[34] MacNeill C, Ayres B, Laverriere AC, Burch JB (1997). Transcripts for functionally distinct isoforms of chicken GATA-5 are differentially expressed from alternative first exons.. J Biol Chem.

[35] Asnagli H, Afkarian M, Murphy KM (2002). Cutting edge: Identification of an alternative GATA-3 promoter directing tissue-specific gene expression in mouse and human.. J Immunol.

[36] Nony P, Hannon R, Gould H, Felsenfeld G (1998). Alternate promoters and developmental modulation of expression of the chicken GATA-2 gene in hematopoietic progenitor cells.. J Biol Chem.

[37] Minegishi N, Ohta J, Suwabe N, Nakauchi H, Ishihara H (1998). Alternative promoters regulate transcription of the mouse GATA-2 gene.. J Biol Chem.

[38] Rojas A, De Val S, Heidt AB, Xu SM, Bristow J (2005). Gata4 expression in lateral mesoderm is downstream of BMP4 and is activated directly by Forkhead and GATA transcription factors through a distal enhancer element.. Development.

[39] Dusing MR, Wiginton DA (2005). Epithelial lineages of the small intestine have unique patterns of GATA expression.. J Mol Histol.

[40] Fang R, Olds LC, Sibley E (2006). Spatio-temporal patterns of intestine-specific transcription factor expression during postnatal mouse gut development.. Gene Expr Patterns.

[41] Ayoubi TA, Van de Ven WJ (1996). Regulation of gene expression by alternative promoters.. FASEB J.

[42] Kimura R, Yoshii H, Nomura M, Kotomura N, Mukai T (2000). Identification of novel first exons in Ad4BP/SF-1 (NR5A1) gene and their tissue- and species-specific usage.. Biochem Biophys Res Commun.

[43] Heicklen-Klein A, Evans T (2004). T-box binding sites are required for activity of a cardiac GATA-4 enhancer.. Dev Biol.

[44] Wang H, Li R, Hu Y (2009). The alternative noncoding exons 1 of aromatase (Cyp19) gene modulate gene expression in a posttranscriptional manner.. Endocrinology.

[45] Bouchard MF, Taniguchi H, Viger RS (2009). The effect of human GATA4 gene mutations on the activity of target gonadal promoters.. J Mol Endocrinol.

[46] Munsick RA (1984). Human fetal extremity lengths in the interval from 9 to 21 menstrual weeks of pregnancy.. Am J Obstet Gynecol.

[47] Shil P, Dudani N, Vidyasagar PB (2006). ISHAN: sequence homology analysis package.. In Silico Biol.

[48] Guigon CJ, Coudouel N, Mazaud-Guittot S, Forest MG, Magre S (2005). Follicular cells acquire sertoli cell characteristics after oocyte loss.. Endocrinology.

[49] Tremblay JJ, Viger RS (2001). GATA factors differentially activate multiple gonadal promoters through conserved GATA regulatory elements.. Endocrinology.

[50] Mazaud S, Guigon CJ, Lozach A, Coudouel N, Forest MG (2002). Establishment of the reproductive function and transient fertility of female rats lacking primordial follicle stock after fetal gamma-irradiation.. Endocrinology.

[51] Matzura O, Wennborg A (1996). RNAdraw: an integrated program for RNA secondary structure calculation and analysis under 32-bit Microsoft Windows.. Comput Appl Biosci.

[52] Schwartz S, Elnitski L, Li M, Weirauch M, Riemer C (2003). MultiPipMaker and supporting tools: Alignments and analysis of multiple genomic DNA sequences.. Nucleic Acids Res.

