# Supplementary figures and images for: Conserved Usage of Alternative 5′ Untranslated Exons of the GATA4 Gene
Source: PLoS One. 2009 Dec 24;4(12):e8454. doi: 10.1371/journal.pone.0008454 (PMC2795200; doi:10.1371/journal.pone.0008454)

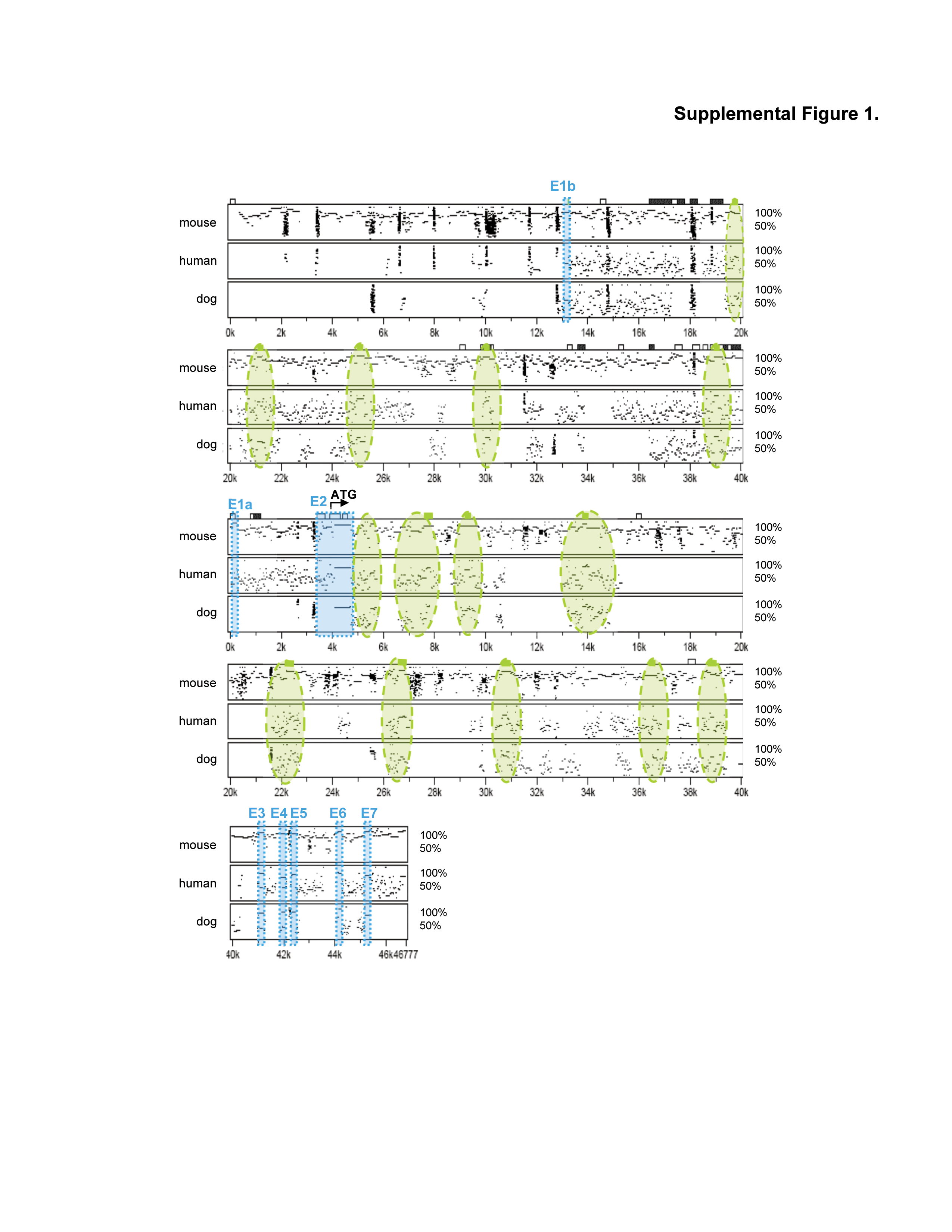

Supplement: Figure S1 — MultiPipMaker [52] sequence comparison of GATA4 rat (used as reference sequence), mouse, human and dog loci. The percent sequence identity per 100 consecutive bp is indicated on the right. Exons are boxed in blue; highly conserved non-coding regions are highlighted by a green oval. (1.63 MB TIF) [file pone.0008454.s001.tif]
